# Food consumption in Brazil: influence of beef on environmental impact and nutritional quality of the diet

**DOI:** 10.11606/s1518-8787.2022056004830

**Published:** 2022-11-18

**Authors:** Josefa Maria Fellegger Garzillo, Vanessa Fadanelli Schoenardie Poli, Fernanda Helena Marrocos Leite, Euridice Martinez Steele, Priscila Pereira Machado, Maria Laura da Costa Louzada, Renata Bertazzi Levy, Carlos Augusto Monteiro

**Affiliations:** I Universidade de São Paulo Faculdade de Saúde Pública Núcleo de Pesquisas Epidemiológicas em Nutrição e Saúde São Paulo SP Brasil Universidade de São Paulo. Faculdade de Saúde Pública. Núcleo de Pesquisas Epidemiológicas em Nutrição e Saúde. São Paulo, SP, Brasil; II Deakin University Institute for Physical Activity and Nutrition Melbourne Australia Deakin University. Institute for Physical Activity and Nutrition. Melbourne, Australia; III Universidade de São Paulo Faculdade de Medicina Departamento de Medicina Preventiva São Paulo SP Brasil Universidade de São Paulo. Faculdade de Medicina. Departamento de Medicina Preventiva. São Paulo, SP, Brasil

**Keywords:** Eating, Meat, Nutrition Assessment, Carbon Footprint, Water Use

## Abstract

**OBJECTIVE:**

To estimate beef consumption and its influence on carbon and water footprints, as well as to improve the nutritional quality of the Brazilian diet.

**METHODS:**

The amount of beef and other foods consumed was evaluated by two 24-hour food records in a representative sample of the Brazilian population ≥ 10 years of age (n = 32,853) from 2008 to 2009. The environmental impact of the diet considered the coefficients of the carbon footprint (gCO_2_ and/kg) and the water footprint (liters/kg) of the foods, as well as their nutritional quality considering the nutrient composition of each food associated with the prevention of nutritional deficiencies or the increase/decrease in chronic disease risk. Linear and logistic regression models, crude and adjusted for sex, age, education, income, region, and area, were used to respectively study the association of fifths of the caloric contribution of beef with the environmental impacts of the diet and inadequate nutrient intake.

**RESULTS:**

Carbon and water footprints and protein, iron, zinc, vitamin B_12_, saturated fat, and sodium contents were higher in the fraction of the diet composed of beef, whereas fiber and added sugar contents were higher in the fraction composed by the other foods. Dietary beef contribution was directly associated with the carbon and water footprints of the diet and the risk of saturated fat and sodium excess, besides fiber insufficiency, inversely associated with the risk of protein, iron, zinc, and vitamin B_12_ insufficiency.

**CONCLUSION:**

Reducing beef consumption in Brazil would also reduce the carbon and water footprints of the diet, as well as the risk of chronic diseases related to food. Therefore, in order not to increase the risk of nutritional deficiencies, monitoring the increased intake of other foods rich in protein, iron, zinc, and vitamin B_12_ is suggested.

## INTRODUCTION

Actions to reduce global greenhouse gas emissions have not been sufficient so far, challenging the climate stability goals defined in the Paris Agreement^1^. This stability will only be achieved with restrictions on fossil fuels and changes in the consumption pattern of populations, including food^2^. Climate impact studies conclude that the strategy of greater power to alleviate climate change by food is promoting diets with lower consumption of animal-based foods, especially beef^3,4^.

Each country has its own food consumption patterns. For example, daily beef consumption in Sweden^5^, Australia^6^, and Argentina^7^ is 50g, 73g, and 135g per person, respectively. These and other variations imply different nutritional impacts for the reduction in beef consumption, which population studies should evaluate, considering all foods consumed by the population^8–10^. If, on the one hand, beef is an important protein, iron, zinc, and vitamin B_12_ source, on the other hand, we found evidence that its consumption can affect the nutritional quality of the diet and increase the risk of chronic non-communicable diseases, including diabetes, cardiovascular diseases, and some types of cancers^11^.

International and national dietary recommendations increasingly emphasize beef consumption reduction, regarding its impact on human and planetary health^12^. An example is the Dietary Guidelines for the Brazilian population, which recommends red meat consumption in only one third of main meals, with the simultaneous increase in the consumption of plant-based, *in natura,* and minimally processed foods, such as vegetables^13^. Given this context, this article aims to describe beef consumption in Brazil and evaluate its association with carbon and water footprints, as well as to analyze the nutritional quality of the Brazilian diet.

## METHODS

### Data Sources

All data on food consumption analyzed in this study come from the personal food consumption assessment module of the Consumer Expenditure Survey conducted by the Brazilian Institute of Geography and Statistics from May 2008 to May 2009 (POF 2008–2009)^14^.

POF 2008–2009 employed a complex cluster sampling plan with geographic and socioeconomic stratification of all census tracts in Brazil, followed by random sector draws in the beginning and random household draws later. The number of sectors drawn in each stratum was proportional to the number of households in the stratum. Each sector's household draw was done by simple random sampling without replacement. The sample covered 55,970 households and the personal food consumption assessment module was applied in a random subsample of 13,569 households (24.3% of the total households studied)^14^.

Interviews conducted in each stratum of the sample were uniformly distributed over the 12 months of research. Residents aged ten years and older from all households selected for the assessment of personal food consumption were invited to complete two 24-hour food records on non-consecutive days (n = 34,003). The participants reported in these records all the food consumed, how it was prepared, and the quantities consumed expressed in homemade measures. Individual data on age, sex, education, family income, and number of people in the household were collected via questionnaires. Household location in urban or rural areas and by Brazilian macro-region completes the record of sociodemographic data available in POF 2008–2009.

Food amounts in homemade measures were converted into grams based on the Table of Reference Measures for Food Consumed in Brazil^15^ and then converted into energy and nutrients based on the Table of Nutritional Composition for Food Consumed in Brazil^16^. Culinary preparations were previously disaggregated in their components according to standardized recipes^17^.

### Data Analysis

All analyses were performed with food consumption data of individuals who completed the two days of the 24-hour food record (n = 32,853), corresponding to 96.8% of the total sample (n = 34,003), always using the daily mean of consumption observed in those two days.

### Evaluation of the Environmental Impact of the Diet Indicators

The potential of environmental impact of the diet was assessed based on its carbon and water footprints. To estimate them, coefficients that quantify, for beef and for all other foods, atmospheric greenhouse gas emissions and the amount of water involved in their production process were used. The carbon footprint coefficient is expressed in grams of carbon dioxide equivalent per kilogram (gCO_2_ and/kg) and the water footprint coefficient, in liters per kilogram (l/kg), both of which are estimated by the life cycle assessment of products methodology^18^.

The coefficients of carbon footprint and water footprint used in this study are described in the publication “Footprints of food and culinary preparations consumed in Brazil”^17^. This publication presents mean environmental impact coefficients calculated based on coefficients estimated by studies published in scientific articles or used in environmental performance reports of products for each food item reported by the people studied by POF 2008-2009, adopting similar food coefficients in the case of foods that did not have available estimates. Regarding culinary preparations, the coefficients took into account all ingredients included in the preparation. The coefficients also consider conversion factors and cooking indexes that respectively account for the removal of inedible parts and the incorporation or loss of water by the cooking effect. In the carbon footprints estimate emissions related to heating food in an oven or stove during preparation were also included.

The carbon and water footprints were estimated by adding the product of the amount consumed of each food item by its respective footprint coefficient, always expressed by the quotient 1,000 kcal (gCO_2_e/1,000 kcal and l/1,000 kcal).

### Assessment of the Nutritional Quality of the Diet

Assessment of the nutritional quality of the diet considered its content in nutrients whose consumption is associated with the prevention of nutritional deficiencies (protein, iron, zinc, and vitamin B_12_) or the increase (added sugar, saturated fat, and sodium) or decrease (fiber) of chronic non-communicable diseases^19^. The daily intake of protein, added sugar, and saturated fat was expressed as a percentage of total energy intake and the intake of the other nutrients was expressed in g, mg, or mcg per 1,000 kcal.

### Beef Consumption in Brazil

Mean beef consumption in Brazil, with confidence intervals of 95%, was described absolutely (kcal/day) and relatively (% total energy) for the population as a whole and according to sex, age (10–19, 20–29, 30–39, 40–49, 50–59 and ≥ 60 years), fifths of *per capit*a family income, years of education (≤ 4, 5–8, 9–12, > 12), and household location (macro-region, rural, or urban area). Beef consumption included fresh meat and viscera, processed meat and ultra-processed beef products, even though they contained plant components (e.g., ‘frozen lasagna’).

### Beef Consumption and Environmental Footprints of the Diet

The relation between beef consumption and environmental footprints of the diet was initially evaluated by comparing the means of carbon and water footprints of the diet fractions composed of beef or all other foods (paired t-test). We then evaluated the association between beef consumption (quintiles of its contribution to total energy intake) and the environmental footprint of the diet through linear regression models and linear trend tests, considering the sociodemographic variables that were associated with beef consumption.

### Beef Consumption and Nutritional Quality of the Diet

The relation between beef consumption and nutritional quality of the diet was studied similarly to that adopted for the analysis of environmental footprints, comparing the average content of protein, iron, zinc, and vitamin B_12_ and added sugar, saturated fat, sodium, and fiber in the diet fractions composed of beef and all other foods (paired t-test), evaluating the association between fifths of beef consumption and diet content in the nutrients studied. The crude and adjusted association between fifths of beef consumption and the prevalence of inadequate intake of each of the studied nutrients was also analyzed, using it for many logistic regression models. Recommendations of the World Health Organization^19^ were used to define adequate levels of protein intake (10–15% of total energy), saturated fat (< 10% of total energy), added sugar (< 10% of total energy), sodium (< 1mg/1,000 kcal), and fiber (≥ 25g/1,000 kcal) as reference values recommended by the Institute of Medicine of the United States (estimated average requirement) according to sex and age group were used to define adequate levels of iron, zinc, and vitamin B_12_^14,20^.

All analyses were performed in the survey module of the Stata*/*SE software version 14.0, which considers the effects of complex sampling allowing the extrapolation of the results for the Brazilian population. P-value < 0.05 was used to identify significant differences or associations.

## RESULTS

Food intake of the Brazilian population aged ten years or more corresponded to a mean daily intake of 1,901 kcal, of which 177 kcal, or 9.4%, came from beef. Total calorie consumption and beef consumption were higher in males, decreased with age and increased with education and income, with no significant difference between people living in urban or rural areas. Total calorie consumption was higher in the North than in other regions of Brazil and beef consumption was higher in the Midwest and the North. The contribution of beef to total calorie consumption was higher in males, increasing with education and income, also being higher in the Midwest and urban areas (Table 1).

**Table 1:** Total food and beef consumption according to sociodemographic variables. Brazilian population aged ten years or more, 2008–2009, (n = 32,853).

Variable		Sample distribution	Total food intake (kcal/day)	Beef consumption^a^	
				Absolute (kcal/day)	Relative to total consumption (%)
		%	mean (95%CI)	mean (95%CI)	mean (95%CI)
Sex					
	Male	48	2,102 (2,061–2,143)	205 (198–212)	9.9 (9.6–10.2)
	Female	52	1,714 (1,696–1,733)^b^	152 (147–156)^b^	9.0 (8.7–9.2)^b^
Age (years)					
	10–19	22	2,017 (1,977–2,057)	179 (170–189)	9.0 (8.6–9.3)
	20–29	21	2,011 (1,977–2,046)	197 (188–206)	9.9 (9.4–10.3)
	30–39	18	1,932 (1,898–1,966)	190 (181–198)	9.9 (9.5–10.4)
	40–49	16	1,851 (1,813–1,889)	183 (173–194)	9.9 (9.4–10.4)
	50–59	12	1,760 (1,720–1,800)	156 (148–164)	9.0 (8.5–9.5)
	≥ 60	13	1,683 (1,570–1,796)^b^	140 (131–148)^b^	8.6 (8.1–9.1)
Education level (years)					
	≤ 4	33	1,769 (1,742–1,795)	159 (152–165)	8.9 (8.6–9.2)
	5–8	27	1,931 (1,896–1,966)	180 (172–187)	9.4 (9.0–9.8)
	9–12	30	1,985 (1,956–2,015)	195 (188–203)	9.9 (9.6–10.3)
	> 12	11	1,999 (1,856–2,142)^b^	180 (168–193)^b^	9.7 (8.9–10.6)^b^
Income (Brazilian reais)					
	7.00–225.27	20	1,784 (1,746–1,822)	151 (143–160)	8.4 (7.9–8.8)
	225.37–399.75	20	1,910 (1,867–1,952)	177 (165–188)	9.3 (8.8–9.8)
	399.77–637.23	20	1,872 (1,832–1,911)	179 (168–190)	9.7 (9.2–10.2)
	637.33–1,151.49	20	1,945 (1,900–1,989)	192 (181–204)	10.0 (9.4–10.5)
	1,151.53–45,879.38	20	1,993 (1,908–2,078)^b^	187 (176–198)^b^	9.8 (9.2–10.5)^b^
Region					
	North	8	2,058 (2,006–2,110)	203 (190–215)^d^	10.0 (9.4–10.5)^c^
	Northeast	28	1,936 (1,875–1,997)^c^	176 (168–185)^c^	9.2 (8.8–9.5)^c^
	Southeast	43	1,863 (1,828–1,897)^c^	168 (158–177)^c^	9.0 (8.6–9.5)^c^
	South	15	1,896 (1,847–1,944)^c^	175 (164–185)^c^	9.3 (8.8–9.7)^c^
	Midwest	7	1,833 (1,759–1,908)^c^	221 (210–231)^d^	12.4 (11.8–13.0)
Area					
	Rural	16	1,923 (1,881–1,965)	173 (160–185)	8.8 (8.4–9.3)
	Urban	84	1,896 (1,868–1,924)	178 (173–184)	9.6 (9.3–9.8)^b^
	Total	100	1,901 (1,876–1,925)	177 (172–182)	9.4 (9.2–9.7)

95%CI: 95% confidence interval.

a Includes consumption of *in natura* and processed beef and ultra-processed beef-based foods.

b p < 0.05 for dichotomous variables and for linear trend < 0.05 in the case of ordinal variables.

c,d p < 0.05 in the Bonferroni test for comparisons of macro-regions two to two when macro-regions do not share the same letter in superscript.

Table 2 presents the estimates of environmental impact indicators and nutritional quality of the Brazilian diet and two fractions of this diet restricted to beef and other foods, respectively. Carbon and water footprints per 1,000 kcal were significantly higher in the beef fraction exceeding by 18 and 11 times, respectively, the footprints observed in the other foods fraction.

**Table 2:** Means (with 95% confidence intervals) of environmental impact and nutritional quality of total food consumption indicators and two fractions of this consumption. Brazilian population aged ten years or more, 2008–2009, (n = 32,853).

Indicators	Total food consumption	Fraction of the consumption relative to:	
		Beef^a^	Other foods
Environmental impact			
Carbon footprint (gCO2e/1,000 kcal)	2,769 (2,732–2,805)	16,547 (16,424–16,669)	916 (904–928)^b^
Water footprint (liters/1,000 kcal)	2,307 (2,281–2,333)	11,584 (11,487–11,680)	1,060 (1,048–1,072)^b^
Nutritional quality			
Protein (% total energy)	16.5 (16.4–16.6)	40.7 (40.4–41.0)	13.2 (13.1–13.3)^b^
Fibers (g/1,000 kcal)	9.3 (9.2–9.4)	0.38 (0.35–0.42)	10.60 (10.49–10.70)^b^
Iron (mg/1,000 kcal)	5.65 (5.61–5.69)	11.66 (11.55–11.76)	4.86 (4.81–4.90)^b^
Zinc (mg/1,000 kcal)	6.04 (5.99–6.09)	23.07 (22.92–23.22)	3.76 (3.72–3.80)^b^
Vitamin B_12_ (mcg/1,000 kcal)	2.94 (2.79–3.09)	17.3 (16.0–18.6)	1.08 (1.05–1.12)^b^
Added sugar (% total energy)	9.1 (8.9–9.3)	0.50 (0.41–0.59)	10.27 (10.06–10.49)^b^
Saturated fat (% total energy)	10.9 (10.8–11.0)	20.6 (20.5–20.7)	9.5 (9.4–9.6)^b^
Sodium (mg/1,000 kcal)	1,440 (1,431–1,449)	2,138 (2,104–2,172)	1,346 (1,337–1,354)^b^

a Includes consumption of *in natura* (fresh meat and viscera) and processed beef and ultra-processed beef-based foods.

b p < 0.05 for the comparison of means between the two fractions.

Significant and equally high-magnitude differences were observed between the beef fraction and the other foods fraction for all studied nutrients. The beef content associated with the prevention of nutritional deficiencies—protein, iron, zinc, and vitamin B_12_—and the content of saturated fat and sodium, nutrients associated with the risk of chronic non-communicable diseases, were higher in the fraction. The added sugar content associated with the risk of chronic diseases and the fiber content associated with the prevention of those diseases were higher in the other foods fraction (Table 2).

Table 3 evaluates the association between beef in the diet (fifths of the contribution of food to total calorie consumption) and indicators of environmental impact and nutritional quality of the diet.

**Table 3:** Means (with 95% confidence intervals) of environmental impact and nutritional quality of food consumption indicators per fifth of beef contribution to total energy intake. Brazilian population aged ten years or more, 2008–2009, (n = 32,853).

Indicators		Estimate	Fifths of beef contribution^a^,^b^				
			Q1	Q2	Q3	Q4	Q5
Environmental impact							
	Carbon footprint (gCO2e/1,000 kcal)	Crude	1,092 (1,057–1,127)	1,589 (1,567–1,611)	2,166 (2,144–2,188)	2,879 (2,851–2,907)	4,259 (4,182–4,335)^d^
		Adjusted^c^	1,086 (1,061–1,111)	1,591 (1,571–1,611)	2,166 (2,144–2,187)	2,880 (2,852–2,907)	4,249 (4,170–4,328)^d^
	Water footprint (liters/1,000 kcal)	Crude	1,179 (1,157–1,200)	1,530 (1,508–1,551)	1,904 (1,882–1,926)	2,369 (2,347–2,391)	3,305 (3,252–3,359)^d^
		Adjusted^c^	1,186 (1,165–1,208)	1,528 (1,507–1,549)	1,901 (1,880–1,922)	2,369 (2,347–2,390)	3,302 (3,247–3,357)^d^
Nutritional quality							
	Protein (% total energy)	Crude	16.7 (16.3–17.0)	15.4 (15.1–15.6)	16.0 (15.7–16.2)	16.5 (16.3–16.7)	17.9 (17.7–18.1)^d^
		Adjusted^c^	16.6 (16.3–16.9)	15.4 (15.2–15.6)	16.0 (15.8–16.2)	16.6 (16.4–16.7)	17.9 (17.7–18.1)^d^
	Fibers (g/1,000 kcal)	Crude	10.2 (10.0–10.4)	9.7 (9.5–9.9)	9.6 (9.5–9.8)	9.4 (9.2–9.5)	8.7 (8.5–8.8)^d^
		Adjusted^c^	10.1 (9.9–10.3)	9.7 (9.6–9.9)	9.6 (9.5–9.8)	9.4 (9.3–9.6)	8.7 (8.6–8.9)^d^
	Iron (mg/1,000 kcal)	Crude	5.0 (4.9–5.1)	5.1 (5.0–5.2)	5.4 (5.3–5.4)	5.7 (5.7–5.8)	6.3 (6.2–6.4)^d^
		Adjusted^c^	5.0 (4.9–5.1)	5.1 (5.1–5.2)	5.4 (5.3–5.4)	5.7 (5.7–5.8)	6.3 (6.2–6.4)^d^
	Zinc (mg/1,000 kcal)	Crude	4.2 (4.1–4.2)	4.7 (4.6–4.8)	5.4 (5.3–5.4)	6.2 (6.1–6.2)	7.8 (7.6–7.9)^d^
		Adjusted^c^	4.2 (4.1–4.2)	4.7 (4.6–4.8)	5.4 (5.3–5.4)	6.2 (6.1–6.2)	7.8 (7.7–7.9)^d^
	Vitamin B_12_ (mcg/1,000 kcal)	Crude	1.8 (1.4–2.1)	1.9 (1.8–2.0)	2.5 (2.3–2.7)	3.0 (2.8–3.3)	4.2 (3.8–4.6)^d^
		Adjusted^c^	1.8 (1.4–2.1)	1.9 (1.8–2.0)	2.5 (2.3–2.7)	3.0 (2.8–3.3)	4.2 (3.8–4.6)^d^
	Added sugar (% total energy)	Crude	8.4 (8.0–8.7)	10.1 (9.7–10.4)	9.4 (9.0–9.7)	9.0 (8.6–9.3)	8.1 (7.8–8.4)^d^
		Adjusted^c^	8.7 (8.4–9.0)	9.8 (9.5–10.2)	9.3 (9.0–9.6)	8.9 (8.6–9.2)	8.1 (7.8–8.4)^d^
	Saturated fat (% total energy)	Crude	9.7 (9.6–9.8)	10.4 (10.1–10.6)	10.5 (10.3–10.6)	10.8 (10.6–11.0)	11.8 (11.7–12.0)^d^
		Adjusted^c^	9.8 (9.7–10.0)	10.3 (10.1–10.5)	10.4 (10.3–10.6)	10.8 (10.6–11.0)	11.8 (11.7–12.0)^d^
	Sodium (mg/1,000 kcal)	Crude	1,435 (1,419–1,451)	1,374 (1,358–1,390)	1,409 (1,395–1,422)	1,441 (1,428–1,454)	1,526 (1,508–1,544)^d^
		Adjusted^c^	1,429 (1,414–1,444)	1,377 (1,362–1,393)	1,410 (1,396–1,423)	1,442 (1,429–1,455)	1,526 (1,508–1,545)^d^

a Includes consumption of *in natura* (fresh meat and viscera) and processed beef and ultra-processed beef-based foods.

b The percentages of beef contribution to the total energy intake in each fifth are: Q = 0.0% (0%-0%); Q2 = 3.7% (0.04%–5.7%); Q3 = 7.7% (5.7%–9.8%); Q4 = 12.7% (9.8%–16.0%); Q5 = 23.6% (16.0%–73.4%).

c Adjusted means for the differences between the fifths in relation to the distribution of the variables sex, age, education, income, region, and area.

d p < 0.05 for linear trend.

Increases in beef consumption lead to a linear and significant increase in the carbon and water footprints of the diet, which are, respectively, three to four times higher among 20% of the population with higher relative beef consumption than among the 20% with lower consumption.

Increases in beef consumption lead to a substantial increase in the diet content of protein, iron, zinc, and vitamin B_12_, and a slight, albeit significant, decrease in added sugar content. Besides, they lead to a substantial reduction in fiber diet content and a substantial increase in saturated fat and sodium content.

Table 4 presents estimates of the prevalence of inadequate nutrient intake in the Brazilian population and evaluates the association of this prevalence with beef consumption (fifths). The prevalence is very high (about 90%) for fiber insufficiency and for sodium excess, high (55.6%) for saturated and moderate fat excess (around 30%), added sugar excess, and for iron, zinc, and vitamin B_12_ insufficiency. Prevalence of protein insufficiency is low (4.2%). Increases in beef consumption lead to substantial reductions in protein, iron, zinc, and vitamin B_12_ insufficiency, and also substantial increases in fiber insufficiency or saturated fat and sodium excess. A statistically significant association was not observed between relative beef consumption and the prevalence of added sugar excess.

**Table 4:** Prevalence (%) and crude and adjusted odds ratio (with 95% confidence intervals) for inadequate nutrient intake per fifth of beef contribution to total energy intake. Brazilian population aged ten years or more, 2008–2009, (n = 32,853).

Nutrients	Prevalence/Odds ratio (OR)	Fifths of the percentage beef contribution^a^,^b^					
		Q1	Q2	Q3	Q4	Q5	Total
Protein	Prevalence	9.1	7.0	2.8	1.4	0.4	4.2
	Crude OR	1 (ref.)	0.75 (0.60–0.95)	0.29 (0.22–0.38)	0.14 (0.10–0.19)	0.04 (0.02–0.07)^d^	
	Adjusted OR^c^	1 (ref.)	0.77 (0.60–0.97)	0.30 (0.23–0.38)	0.14 (0.10–0.20)	0.04 (0.02–0.07)^d^	
Fibers	Prevalence	77.1	84.2	84.2	86.8	90.7	84.3
	Crude OR	1 (ref.)	1.58 (1.33–1.88)	1.58 (1.35–1.85)	1.94 (1.65–2.29)	2.89 (2.42–3.46)^d^	
	Adjusted OR^c^	1 (ref.)	1.43 (1.20–1.70)	1.45 (1.23–1.71)	1.76 (1.49–2.07)	2.65 (2.20 –3.18)^d^	
Iron	Prevalence	39.8	20.7	23.8	20.7	18.3	25.4
	Crude OR	1 (ref.)	0.39 (0.34–0.45)	0.47 (0.42–0.54)	0.40 (0.35–0.46)	0.34 (0.30–0.39)^d^	
	Adjusted OR^c^	1 (ref.)	0.37 (0.32–0.43)	0.45 (0.39–0.52)	0.39 (0.33–0.45)	0.33 (0.29–0.39)^d^	
Zinc	Prevalence	68.1	36.6	30.9	22.5	14.4	35.6
	Crude OR	1 (ref.)	0.27 (0.24–0.31)	0.21 (0.18–0.24)	0.14 (0.12–0.16)	0.08 (0.07–0.09)^d^	
	Adjusted OR^c^	1 (ref.)	0.28 (0.24–0.32)	0.21 (0.18–0.24)	0.13 (0.12–0.15)	0.07 (0.06–0.09)^d^	
Vitamin B^12^	Prevalence	58.2	31.8	22.0	14.2	9.4	29
	Crude OR	1 (ref.)	0.33 (0.29–0.38)	0.20 (0.18–0.23)	0.12 (0.10–0.14)	0.07 (0.06–0.09)^d^	
	Adjusted OR^c^	1 (ref.)	0.34 (0.30–0.39)	0.20 (0.17–0.23)	0.12 (0.10–0.13)	0.08 (0.06–0.09)^d^	
Added sugar	Prevalence	33.8	43.5	40.6	38.7	34.5	37.9
	Crude OR	1 (ref.)	1.50 (1.32–1.72)	1.34 (1.17–1.52)	1.24 (1.08–1.41)	1.03 (0.89–1.19)	
	Adjusted OR^c^	1 (ref.)	1.32 (1.14–1.52)	1.21 (1.05–1.39)	1.10 (0.96–1.26)	0.92 (0.79–1.07)	
Saturated fat	Prevalence	43.0	48.7	52.8	58.6	76.0	55.6
	Crude OR	1 (ref.)	1.26 (1.10–1.43)	1.48 (1.31–1.68)	1.88 (1.65–2.14)	4.21 (3.63–4.88)^d^	
	Adjusted OR^c^	1 (ref.)	1.16 (1.02–1.33)	1.41 (1.24–1.60)	1.83 (1.60–2.08)	4.38 (3.75–5.11)^d^	
Sodium	Prevalence	90.8	88.5	90.6	93.4	96.6	91.8
	Crude OR	1 (ref.)	0.78 (0.64–0.95)	0.99 (0.81–1.20)	1.44 (1.14–1.83)	2.91 (2.26–3.74)^d^	
	Adjusted OR^c^	1 (ref.)	0.79 (0.64–0.96)	0.99 (0.81–1.20)	1.43 (1.13–1.82)	2.91 (2.26–3.74)^d^	

a Includes consumption of *in natura* (fresh meat and viscera) and processed beef and ultra-processed beef-based foods

b The percentages of beef contribution to the total energy intake in each fifth are: Q = 0.0% (0%-0%); Q2 = 3.7% (0.04%–5.7%); Q3 = 7.7% (5.7%–9.8%); Q4 = 12.7% (9.8%–16.0%); Q5 = 23.6% (16.0%–73.4%).

c Adjusted odds ratio for differences between the fifths in relation to the distribution of the variables sex, age, education, income, region, and area.

d p < 0.05 for linear trend.

## DISCUSSION

Based on the food intake of a representative sample of the Brazilian population aged ten years or older (n = 32,853)^14^, our study estimated that beef consumption in the Brazilian diet corresponds to 9.4% of the total energy intake. This consumption is higher among men, increases with income level and education and is higher among residents of the Midwest and urban areas. The carbon and water footprints of the diet increase linearly with fifths of beef consumption, being three to four times higher among the 20% with higher consumption than among the 20% with lower consumption. The same increase in beef consumption produces mixed results in the nutritional quality of the diet. On the one hand, it increases its protein, iron, zinc, and vitamin B_12_ content, substantially reducing the prevalence of insufficient intake of these nutrients—which is low in the case of protein and moderate in the case of micronutrients. On the other hand, it reduces the fiber content of the diet and increases its saturated fat and sodium content, increasing the high or very high prevalence of inadequate intake of these three nutrients.

We did not find any studies in the literature that have contrasted the relation that beef consumption maintains with the environmental footprints of the diet and with its nutritional quality. We found, however, several studies that describe the association of the carbon footprint of food or dietary patterns with its nutritional quality, evidencing in general a heterogeneous pattern of association. A systematic review of these studies, mostly conducted in high-income countries, indicates that foods and dietary patterns with low carbon footprint promote a nutritional quality of the diet, as they are associated with lower saturated fat content, but act differently when the content of micronutrients such as iron, zinc, and vitamin B_12_^9^ is reduced. These results are consistent with our study data.

Given so many variables, it is still possible to find diets that fit nutritional adequacy and alleviate environmental impacts. The Mediterranean and new Nordic diets—both with vegetable predominance and low meat consumption—are examples of healthy eating patterns that contribute to alleviating environmental impacts^21^. The adoption of food standards recommended by the Dutch dietary guidelines offers the possibility of reducing all causes of mortality, while moderately reducing climate impacts^22^. A study of the average Brazilian diet (POF 2008/09) adjusted to the recommendations of the Dietary Guidelines for the Brazilian population^14^ showed the possibility of reducing carbon footprint by 14.8% and water footprint by 18.0%^23^.

Measures to restrict beef consumption should be done cautiously, since it is a source of essential micronutrients. Our study found an upward trend in inadequate iron, zinc, and vitamin B_12_ consumption the lower the beef consumption. Replacing all meat and dairy products with plant-based foods in the diet of children in the Netherlands resulted in a decrease of 5% to 13% in calcium, zinc, and vitamin B_12_ intake and 49% in vitamin B_12_ intake, besides increasing iron intake with lower bioavailability^24^.

The relationship between beef consumption (and other animal-based foods) and micronutrient content in the diet serves as a warning so that the nutritional quality of food is always considered when characterizing environmentally sustainable diets. For beef reduction recommendations to be consistent with climate impact alleviation, nutritional challenges cannot be overlooked.

The intensity of the environmental impact of food is one of several criteria to be used in dietary choices. The nutritional quality of the foods that make up the plant fraction of diets is important, since not all plant-based foods have beneficial cardiovascular effects and even some of them may be harmful to health, as is the case of ultra-processed plant-based foods^25^. This means that such foods should be avoided regardless of their high or low environmental impact potential.

Reduction of beef consumption by the Brazilian population is a key strategy in alleviating climate impacts in the country, but its effectiveness is limited. Although beef consumption in Brazil decreases, the country is the main beef supplier in the international market. Environmental sustainability of the food system cannot simply depend on changes in domestic demand for beef; the increase in volume of beef exports or interruptions in slaughter could annul possible positive environmental results achieved by lower domestic consumption^26,27^. The transformations in livestock production that result in low environmental impact production systems are urgent.

This study's strong points include a representative sample of the Brazilian population, a food consumption measurement by the record of all foods in two 24-hour periods, and an evaluation of the impact of beef consumption on the environment and the nutritional quality of the diet, enabling diet evaluations in real consumption situations in different population strata. This study focused on beef, a relevant food in Brazil's culinary culture, whose productive sector is responsible for about 25% of the greenhouse gas emissions recorded in the national inventory^28^. The comprehensive assessment of the nutritional quality of diets in parallel with measures of inadequate consumption of critical nutrients and others that should be limited was an innovation of our research.

One of the main limitations of this study concerns the use of carbon and water footprint coefficients estimated not always based on the production conditions observed in Brazil. Another limitation is the inaccuracy in quantifying food and nutrient consumption due to self-reported food intake and the use of nutritional composition tables of foods that do not always translate the composition of the foods ingested by the participants. The environmental footprints of ultra-processed beef products were considered for the product as a whole, consisting of other plant-based ingredients and additives, and not only for the beef portion. Finally, the non-inclusion of children under ten years of age in the sample studied makes it impossible to generalize results for this age group.

Another limitation is based on a food consumption survey conducted more than ten years ago. However, by comparing the estimated consumption by the POF carried out in 2008/09^14^ with that estimated by the most recent POF of 2017/18,^29^ we observe minute changes, including beef consumption: 63 g/person/day and 60 g/person/day, respectively.

This study aimed to show some nutritional and environmental influences of beef consumption in Brazilian diets. Recent frameworks of reduced beef consumption, food insecurity, and hunger due to an impoverishing Brazilian population—parallel with the food production and deforestation records—only confirm the unsustainability of the Brazilian food system. We reiterate that a sustainable food system assumes the population's food security and must be evaluated in the various dimensions of sustainable development: social, cultural, environmental, ecological, territorial, economic, and political^30^.

## CONCLUSION

Reduction in beef consumption in Brazil would cause the carbon and water footprints of the diet and the risk of chronic diseases related to food to decrease, but in order not to increase the risk of nutritional deficiencies, the reduction would have to be accompanied by an increased intake of other foods rich in protein, iron, zinc, and vitamin B_12_.
