# Supplementary material for: Food consumption in Brazil: influence of beef on environmental impact and nutritional quality of the diet
Source: Rev Saude Publica. 2022 Nov 18;56:102. doi: 10.11606/s1518-8787.2022056004830 (PMC9749736; doi:10.11606/s1518-8787.2022056004830)
Supplement: Translation

# Translation: Consumo alimentar no Brasil: influência da carne bovina no impacto ambiental e na qualidade nutricional da dieta

**Authors:** Josefa Maria Fellegger Garzillo, Vanessa Fadanelli Schoenardie Poli, Fernanda Helena Marrocos Leite, Euridice Martinez Steele, Priscila Pereira Machado, Maria Laura da Costa Louzada, Renata Bertazzi Levy, Carlos Augusto Monteiro

**Affiliations:** I São Paulo SP Brasil Universidade de São Paulo. Faculdade de Saúde Pública. Núcleo de Pesquisas Epidemiológicas em Nutrição e Saúde. São Paulo, SP, Brasil; II Melbourne Australia Deakin University. Institute for Physical Activity and Nutrition. Melbourne, Australia; III São Paulo SP Brasil Universidade de São Paulo. Faculdade de Medicina. Departamento de Medicina Preventiva. São Paulo, SP, Brasil

**Keywords:** Ingestão de Alimentos, Carne, Avaliação Nutricional, Pegada de Carbono, Usos da Água

## Abstract

**OBJETIVO:**

Estimar o consumo de carne bovina e a sua influência nas pegadas de carbono e na pegada hídrica, bem como mesurar a qualidade nutricional da dieta no Brasil.

**MÉTODOS:**

A quantidade consumida de carne bovina e dos demais alimentos foi avaliada por dois registros alimentares de 24 horas em amostra representativa da população brasileira ≥ 10 anos de idade (n = 32.853) entre 2008 e 2009. O impacto ambiental da dieta considerou os coeficientes da pegada de carbono (gCO_2_e/kg) e da pegada hídrica (litros/kg) dos alimentos, bem como sua qualidade nutricional considerando a composição de cada alimento em nutrientes associados à prevenção de deficiências nutricionais ou ao aumento/diminuição do risco de doenças crônicas. Modelos de regressão linear e logística, brutos e ajustados para sexo, idade, escolaridade, renda, região e área, foram utilizados para estudar, respectivamente, a associação de quintos da contribuição calórica de carne bovina com os impactos ambientais da dieta e com a ingestão inadequada de nutrientes.

**RESULTADOS:**

As pegadas de carbono e hídrica e os teores de proteína, ferro, zinco, vitamina B_12_, gordura saturada e sódio foram maiores na fração da dieta composta por carnes bovinas, enquanto o teor de fibra e de açúcar de adição foram maiores na fração composta pelos demais alimentos. A contribuição dietética de carne bovina mostrou-se associada diretamente com as pegadas de carbono e hídrica da dieta e com o risco de ingestão excessiva de gordura saturada e de sódio, além de ingestão insuficiente de fibra, associando-se inversamente com o risco de ingestão insuficiente de proteína, ferro, zinco e vitamina B_12_.

**CONCLUSÃO:**

A redução no consumo de carne bovina no Brasil diminuiria as pegadas de carbono e hídrica da dieta, assim como o risco de doenças crônicas relacionadas à alimentação. Portanto, para não aumentar o risco de deficiências nutricionais, é sugerido o acompanhamento do aumento da ingestão de outros alimentos fontes de proteína, ferro, zinco e vitamina B_12_.

## INTRODUÇÃO

As ações para redução das emissões globais de gases do efeito estufa não se mostraram suficientes até o momento, desafiando as metas para estabilidade climática estipuladas no Acordo de Paris^1^. Essa estabilidade apenas será alcançada com restrições ao uso dos combustíveis fósseis e modificações no padrão de consumo das populações, incluindo a alimentação^2^. Os estudos de impacto climático concluem que a estratégia de maior poder de mitigação das mudanças climáticas, por meio da alimentação é promovendo dietas com menor participação de alimentos de origem animal, sobretudo de carne bovina^3–4^.

De um país para outro existem variações nos padrões de consumo alimentar. Por exemplo, o consumo diário de carne bovina na Suécia^5^, na Austrália^6^ e na Argentina^7^ é de 50g, de 73g e 135g por pessoa, respectivamente. Essas e outras variações implicam impactos nutricionais diferenciados para a redução no consumo de carne bovina, que devem ser avaliados por estudos populacionais que considerem o conjunto dos alimentos consumidos pela população^8–10^. Se, por um lado, a carne bovina é uma importante fonte de proteína, ferro, zinco e vitamina B_12_, por outro, há evidências de que seu consumo pode afetar a qualidade nutricional da dieta e aumentar o risco de doenças crônicas não transmissíveis, incluindo diabetes, doenças cardiovasculares e alguns tipos de cânceres^11^.

Cada vez mais, as recomendações alimentares internacionais e nacionais enfatizam a diminuição do consumo de carne bovina, tendo em vista seu impacto sobre a saúde humana e planetária^12^. Um exemplo é o Guia Alimentar para a População Brasileira, que recomenda o consumo de carnes vermelhas em apenas um terço das refeições principais, com o concomitante aumento do consumo de alimentos de origem vegetal, *in natura* e minimamente processados, como os legumes e as verduras^13^. Nesse contexto, este artigo tem o objetivo de descrever o consumo da carne bovina no Brasil e avaliar sua associação com a pegada de carbono e a pegada hídrica, além de analisar a qualidade nutricional da dieta brasileira.

## MÉTODOS

### Fontes de Dados

Todos os dados sobre consumo alimentar analisados neste estudo são provenientes do módulo de avaliação do consumo alimentar pessoal da Pesquisa de Orçamentos Familiares realizada pelo Instituto Brasileiro de Geografia e Estatística entre maio de 2008 e maio de 2009 (POF 2008–2009)^14^.

A POF 2008–2009 empregou um plano amostral complexo, por conglomerados, com estratificação geográfica e socioeconômica de todos os setores censitários do país, seguido de sorteios aleatórios de setores em um primeiro estágio e de domicílios em um segundo. O número de setores sorteados em cada estrato foi proporcional ao número de domicílios no estrato. O sorteio dos domicílios de cada setor foi feito por amostragem aleatória simples sem reposição. A amostra abrangeu 55.970 domicílios e o módulo de avaliação do consumo alimentar pessoal foi aplicado em uma subamostra aleatória de 13.569 domicílios (24,3% do total de domicílios estudados)^14^.

As entrevistas realizadas em cada estrato da amostra foram distribuídas uniformemente ao longo dos 12 meses de realização da pesquisa. Os moradores com dez ou mais anos de idade de todos os domicílios sorteados para a avaliação do consumo alimentar pessoal foram convidados a preencherem dois registros alimentares de 24 horas, em dias não consecutivos (n = 34.003). Nesses registros, os participantes relatavam todos os alimentos consumidos, o tipo de preparação e as quantidades consumidas expressas na forma de medidas caseiras. Dados individuais sobre idade, sexo e escolaridade, renda familiar e número de pessoas no domicílio foram coletados por meio de questionários. A localização do domicílio em áreas urbanas ou rurais e por macrorregião do Brasil completa o rol de dados sociodemográficos disponíveis na POF 2008–2009.

As quantidades de alimentos em medidas caseiras foram convertidas em gramas com base na Tabela de Medidas Referidas para os Alimentos Consumidos no Brasil^15^ e, em seguida, convertidas em energia e nutrientes com base na Tabela de Composição Nutricional dos Alimentos Consumidos no Brasil^16^. Preparações culinárias foram previamente desagregadas em seus componentes conforme receitas padronizadas^17^.

### Análise de Dados

Todas as análises deste estudo foram realizadas com os dados de consumo alimentar dos indivíduos que preencheram os dois dias de registro alimentar de 24 horas (n = 32.853), o que correspondeu a 96,8% da amostra total (n = 34.003), utilizando-se sempre a média diária do consumo observado nos dois dias.

### Avaliação dos Indicadores de Impacto Ambiental da Dieta

O potencial de impacto ambiental da dieta foi avaliado com base nas suas pegadas de carbono e hídrica. Para estimar essas pegadas foram usados coeficientes que quantificam, para a carne bovina e para todos os demais alimentos, as emissões atmosféricas de gases do efeito estufa e a quantidade de água envolvida em seu processo produtivo. O coeficiente da pegada de carbono é expresso em gramas de dióxido de carbono equivalente por quilograma (gCO_2_e/kg) e o coeficiente da pegada hídrica em litros por quilograma (l/kg), sendo ambos calculados pela metodologia de avaliação do ciclo de vida de produtos^18^.

Os coeficientes da pegada de carbono e da pegada hídrica utilizados neste estudo são aqueles descritos na publicação “Pegadas dos alimentos e preparações culinárias consumidos o Brasil”^17^. Essa publicação apresenta, para cada item alimentar relatado pelas pessoas estudadas pela POF 2008-2009, coeficientes médios de impacto ambiental calculados com base em coeficientes estimados por estudos publicados em artigos científicos ou utilizados em relatórios de desempenho ambiental de produtos, adotando-se coeficientes de alimentos similares no caso de alimentos que não contavam com estimativas disponíveis. No caso de preparações culinárias, os coeficientes levaram em conta todos os ingredientes incluídos na preparação. Os coeficientes consideram, ainda, fatores de conversão e índices de cocção que contabilizam, respectivamente, a retirada de partes não comestíveis e a incorporação ou perda de água pelo efeito do cozimento. Além disso, no cálculo das pegadas de carbono, incluíram-se as emissões relativas ao aquecimento em forno ou fogão durante o preparo dos alimentos.

As pegadas de carbono e hídrica da dieta foram calculadas somando o produto da quantidade consumida de cada item alimentar pelo seu respectivo coeficiente de pegada, sendo sempre expressas pelo quociente 1.000 kcal (gCO_2_e/1.000 kcal e l/1.000 kcal).

### Avaliação da Qualidade Nutricional da Dieta

A avaliação da qualidade nutricional da dieta considerou seu teor em nutrientes cujo consumo é associado à prevenção de deficiências nutricionais (proteína, ferro, zinco e vitamina B_12_) ou ao aumento (açúcar de adição, gordura saturada, sódio) ou diminuição (fibra) no risco de doenças crônicas não transmissíveis^19^. A ingestão diária de proteína, açúcar de adição e gordura saturada foi expressa como percentual de ingestão total de energia e a ingestão dos demais nutrientes foram expressas em g, mg ou mcg por 1.000 kcal.

### Consumo de Carne Bovina no Brasil

O consumo médio de carne bovina no Brasil, com intervalos de confiança de 95%, foi descrito de forma absoluta (kcal/dia) e relativa (% energia total) para a população como um todo e segundo sexo, idade (10–19, 20–29, 30–39, 40–49, 50–59 e ≥ 60 anos), quintos da renda familiar *per capit*a, anos de escolaridade (≤ 4, 5–8, 9–12, > 12) e localização do domicílio (macrorregião, área rural ou urbana). O consumo de carne bovina incluiu carnes e vísceras frescas, carnes processadas e produtos ultraprocessados à base de carne bovina, ainda que estes contivessem componentes vegetais (por exemplo, “lasanha congelada”).

### Consumo de Carne Bovina e Pegadas Ambientais da Dieta

A relação entre consumo de carne bovina e as pegadas ambientais da dieta foi avaliada inicialmente comparando-se as médias das pegadas de carbono e hídrica das frações da dieta compostas por carne bovina ou por todos os demais alimentos (teste de t pareado). A seguir, avaliou-se a associação entre contribuição da carne bovina (quintos crescentes da sua contribuição para ingestão total de energia) e a pegada ambiental da dieta por meio de modelos de regressão linear e testes de tendência linear, levando-se em conta na análise ajustada as variáveis sociodemográficas que se mostraram associadas ao consumo de carne bovina.

### Consumo de Carne Bovina e Qualidade Nutricional da Dieta

A relação entre consumo de carne bovina e a qualidade nutricional da dieta foi estudada de forma semelhante à adotada para análise das pegadas ambientais, comparando-se o teor médio de proteína, ferro, zinco e vitamina B_12_, açúcar de adição, gordura saturada, sódio e fibra nas frações da dieta compostas por carne bovina e por todos os demais alimentos (teste de t pareado), avaliando-se a associação entre quintos da contribuição da carne bovina e o teor da dieta nos nutrientes estudados. Adicionalmente, foi analisada a associação bruta e ajustada entre quintos da contribuição da carne bovina e a prevalência de ingestão inadequada de cada um dos nutrientes estudados, empregando-se para tantos modelos de regressão logística. Recomendações da Organização Mundial de Saúde^19^ foram empregadas na definição de teores adequados da ingestão de proteína (10–15% do total de energia), gordura saturada (< 10% do total de energia), açúcar de adição (< 10% do total de energia), sódio (< 1mg/1.000 kcal) e fibra ≥ 25g/1.000 kcal) enquanto valores de referência recomendados pelo *Institute of Medicine dos Estado Unidos* (necessidade média estimada, *estimated average requirement*) de acordo com o sexo e a faixa etária foram utilizados na definição de teores adequados de ferro, zinco e vitamina B_12_^14,20^.

Todas as análises foram realizadas no módulo *survey* do software Stata*/*SE versão 14.0, que considera os efeitos da amostragem complexa permitindo a extrapolação dos resultados para a população brasileira. Utilizou-se o valor de p < 0,05 para identificar diferenças ou associações significativas.

## RESULTADOS

O consumo alimentar da população brasileira com dez ou mais anos de idade correspondeu a uma ingestão média diária de 1.901 kcal, sendo 177 kcal, ou 9,4%, provenientes de carne bovina. O consumo total de calorias e o consumo de carne bovina foram maiores no sexo masculino, diminuíram com a idade e aumentaram com a escolaridade e com a renda, não havendo diferença significativa entre pessoas habitadas em áreas urbanas ou rurais. O consumo total de calorias foi maior na região Norte do que nas demais regiões do Brasil e o consumo de carne bovina foi maior nas regiões Centro-Oeste e Norte. A contribuição de carne bovina para o consumo total de calorias foi maior no sexo masculino, aumentando com a escolaridade e a renda, além de ser maior na região Centro-Oeste e nas áreas urbanas (Tabela 1).

**Tabela 1:** Consumo alimentar total e consumo de carne bovina segundo variáveis sociodemográficas. População brasileira com dez ou mais anos de idade, 2008–2009, (n = 32.853).

Variável		Distribuição da amostra	Consumo alimentar total (kcal/dia)	Consumo de carne bovina^a^	
				Absoluto (kcal/dia)	Relativo ao consumo total (%)
		%	média (IC95%)	média (IC95%)	média (IC95%)
Sexo					
	Masculino	48	2.102 (2.061–2.143)	205 (198–212)	9,9 (9,6–10,2)
	Feminino	52	1.714 (1.696–1.733)^b^	152 (147–156)^b^	9,0 (8,7–9,2)^b^
Idade (anos)					
	10–19	22	2.017 (1.977–2.057)	179 (170–189)	9,0 (8,6–9,3)
	20–29	21	2.011 (1.977–2.046)	197 (188–206)	9,9 (9,4–10,3)
	30–39	18	1.932 (1.898–1.966)	190 (181–198)	9,9 (9,5–10,4)
	40–49	16	1.851 (1.813–1.889)	183 (173–194)	9,9 (9,4–10,4)
	50–59	12	1.760 (1.720–1.800)	156 (148–164)	9,0 (8,5–9,5)
	≥ 60	13	1.683 (1.570–1.796)^b^	140 (131–148)^b^	8,6 (8,1–9,1)
Escolaridade (anos)					
	≤ 4	33	1.769 (1.742–1.795)	159 (152–165)	8,9 (8,6–9,2)
	5–8	27	1.931 (1.896–1.966)	180 (172–187)	9,4 (9,0–9,8)
	9–12	30	1.985 (1.956–2.015)	195 (188–203)	9,9 (9,6–10,3)
	> 12	11	1.999 (1.856–2.142)^b^	180 (168–193)^b^	9,7 (8,9–10,6)^b^
Renda (reais)					
	7,00–225,27	20	1.784 (1.746–1.822)	151 (143–160)	8,4 (7,9–8,8)
	225,37–399,75	20	1.910 (1.867–1.952)	177 (165–188)	9,3 (8,8–9,8)
	399,77–637,23	20	1.872 (1.832–1.911)	179 (168–190)	9,7 (9,2–10,2)
	637,33–1.151,49	20	1.945 (1.900–1.989)	192 (181–204)	10,0 (9,4–10,5)
	1.151,53–45.879,38	20	1.993 (1.908–2.078)^b^	187 (176–198)^b^	9,8 (9,2–10,5)^b^
Região					
	Norte	8	2.058 (2.006–2.110)	203 (190–215)^d^	10,0 (9,4–10,5)^c^
	Nordeste	28	1.936 (1.875–1.997)^c^	176 (168–185)^c^	9,2 (8,8–9,5)^c^
	Sudeste	43	1.863 (1.828–1.897)^c^	168 (158–177)^c^	9,0 (8,6–9,5)^c^
	Sul	15	1.896 (1.847–1.944)^c^	175 (164–185)^c^	9,3 (8,8–9,7)^c^
	Centro-Oeste	7	1.833 (1.759–1.908)^c^	221 (210–231)^d^	12,4 (11,8–13,0)
Área					
	Rural	16	1.923 (1.881–1.965)	173 (160–185)	8,8 (8,4–9,3)
	Urbana	84	1.896 (1.868–1.924)	178 (173–184)	9,6 (9,3–9,8)^b^
	Total	100	1.901 (1.876–1.925)	177 (172–182)	9,4 (9,2–9,7)

IC95%: intervalo de confiança de 95%.

a Inclui consumo de carne bovina in natura, processada e alimentos ultraprocessados à base de carne bovina.

b p < 0,05 para variáveis dicotômicas e para tendência linear < 0,05 no caso de variáveis ordinais.

c,d p < 0,05 no teste de Bonferroni para comparações das macrorregiões duas a duas quando as macrorregiões não compartilham a mesma letra em sobrescrito.

A Tabela 2 apresenta estimativas de indicadores do impacto ambiental e da qualidade nutricional da dieta brasileira e de duas frações dessa dieta restritas à carne bovina e aos demais alimentos, respectivamente. As pegadas de carbono e hídrica por 1.000 kcal foram significativamente maiores na fração carne bovina excedendo em 18 e 11 vezes, respectivamente, as pegadas observadas na fração demais alimentos.

**Tabela 2:** Médias (com intervalos de confiança de 95%) de indicadores do impacto ambiental e da qualidade nutricional do consumo alimentar total e de duas frações desse consumo. População brasileira com dez ou mais anos de idade, 2008–2009, (n = 32.853).

Indicadores	Consumo alimentar total	Fração do consumo relativa a:	
		Carne bovina^a^	Demais alimentos
Impacto ambiental			
Pegada de carbono (gCO2e/1.000 kcal)	2.769 (2.732–2.805)	16.547 (16.424–16.669)	916 (904–928)^b^
Pegada hídrica (litros/1.000 kcal)	2.307 (2.281–2.333)	11.584 (11.487–11.680)	1.060 (1.048–1.072)^b^
Qualidade nutricional			
Proteína (% energia total)	16,5 (16,4–16,6)	40,7 (40,4–41,0)	13,2 (13,1–13,3)^b^
Fibras (g/1.000 kcal)	9,3 (9,2–9,4)	0,38 (0,35–0,42)	10,60 (10,49–10,70)^b^
Ferro (mg/1.000 kcal)	5,65 (5,61–5,69)	11,66 (11,55–11,76)	4,86 (4,81–4,90)^b^
Zinco (mg/1.000 kcal)	6,04 (5,99–6,09)	23,07 (22,92–23,22)	3,76 (3,72–3,80)^b^
Vitamina B^12^ (mcg/1.000 kcal)	2,94 (2,79–3,09)	17,3 (16,0–18,6)	1,08 (1,05–1,12)^b^
Açúcar de adição (% energia total)	9,1 (8,9–9,3)	0,50 (0,41–0,59)	10,27 (10,06–10,49)^b^
Gordura saturada (% energia total)	10,9 (10,8–11,0)	20,6 (20,5–20,7)	9,5 (9,4–9,6)^b^
Sódio (mg/1.000 kcal)	1.440 (1.431–1.449)	2.138 (2.104–2.172)	1.346 (1.337–1.354)^b^

a Inclui consumo de carne bovina *in natura* (carnes e vísceras frescas), processada e alimentos ultraprocessados à base de carne bovina.

b p < 0,05 para a comparação de médias entre as duas frações.

Diferenças significativas e igualmente de alta magnitude foram observadas entre a fração carne bovina e a fração demais alimentos quanto a todos os nutrientes estudados. Foram superiores na fração carne bovina tanto o teor dos nutrientes associados à prevenção de deficiências nutricionais – proteína, ferro, zinco e vitamina B_12_ – quanto o teor de gordura saturada e sódio, nutrientes associados ao risco de doenças crônicas não transmissíveis. Já o teor em açúcar de adição associado ao risco de doenças crônicas, e o teor em fibra associado à prevenção daquelas doenças foram superiores na fração demais alimentos (Tabela 2).

A Tabela 3 avalia a associação entre a participação de carne bovina na dieta (quintos da contribuição do alimento para o consumo total de calorias) e indicadores do impacto ambiental e da qualidade nutricional da dieta.

**Tabela 3:** Médias (com intervalos de confiança de 95%) de indicadores do impacto ambiental e da qualidade nutricional do consumo alimentar por quintos da contribuição de carne bovina para a ingestão total de energia. População brasileira com dez ou mais anos de idade, 2008–2009, (n = 32.853).

Indicadores		Estimativa	Quintos da contribuição da carne bovina^a^,^b^				
			Q1	Q2	Q3	Q4	Q5
Impacto ambiental							
	Pegada de carbono (gCO2e/1.000 kcal)	Bruta	1.092 (1.057–1.127)	1.589 (1.567–1.611)	2.166 (2.144–2.188)	2.879 (2.851–2.907)	4.259 (4.182–4.335)^d^
		Ajustada^c^	1.086 (1.061–1.111)	1.591 (1.571–1.611)	2.166 (2.144–2.187)	2.880 (2.852–2.907)	4.249 (4.170–4.328)^d^
	Pegada hídrica (litros/1.000 kcal)	Bruta	1.179 (1.157–1.200)	1.530 (1.508–1.551)	1.904 (1.882–1.926)	2.369 (2.347–2.391)	3.305 (3.252–3.359)^d^
		Ajustada^c^	1.186 (1.165–1.208)	1.528 (1.507–1.549)	1.901 (1.880–1.922)	2.369 (2.347–2.390)	3.302 (3.247–3.357)^d^
Qualidade nutricional							
	Proteína (% energia total)	Bruta	16,7 (16,3–17,0)	15,4 (15,1–15,6)	16,0 (15,7–16,2)	16,5 (16,3–16,7)	17,9 (17,7–18,1)^d^
		Ajustada^c^	16,6 (16,3–16,9)	15,4 (15,2–15,6)	16,0 (15,8–16,2)	16,6 (16,4–16,7)	17,9 (17,7–18,1)^d^
	Fibras (g/1.000 kcal)	Bruta	10,2 (10,0–10,4)	9,7 (9,5–9,9)	9,6 (9,5–9,8)	9,4 (9,2–9,5)	8,7 (8,5–8,8)^d^
		Ajustada^c^	10,1 (9,9–10,3)	9,7 (9,6–9,9)	9,6 (9,5–9,8)	9,4 (9,3–9,6)	8,7 (8,6–8,9)^d^
	Ferro (mg/1.000 kcal)	Bruta	5,0 (4,9–5,1)	5,1 (5,0–5,2)	5,4 (5,3–5,4)	5,7 (5,7–5,8)	6,3 (6,2–6,4)^d^
		Ajustada^c^	5,0 (4,9–5,1)	5,1 (5,1–5,2)	5,4 (5,3–5,4)	5,7 (5,7–5,8)	6,3 (6,2–6,4)^d^
	Zinco (mg/1.000 kcal)	Bruta	4,2 (4,1–4,2)	4,7 (4,6–4,8)	5,4 (5,3–5,4)	6,2 (6,1–6,2)	7,8 (7,6–7,9)^d^
		Ajustada^c^	4,2 (4,1–4,2)	4,7 (4,6–4,8)	5,4 (5,3–5,4)	6,2 (6,1–6,2)	7,8 (7,7–7,9)^d^
	Vitamina B_12_ (mcg/1.000 kcal)	Bruta	1,8 (1,4–2,1)	1,9 (1,8–2,0)	2,5 (2,3–2,7)	3,0 (2,8–3,3)	4,2 (3,8–4,6)^d^
		Ajustada^c^	1,8 (1,4–2,1)	1,9 (1,8–2,0)	2,5 (2,3–2,7)	3,0 (2,8–3,3)	4,2 (3,8–4,6)^d^
	Açúcar de adição (% energia total)	Bruta	8,4 (8,0–8,7)	10,1 (9,7–10,4)	9,4 (9,0–9,7)	9,0 (8,6–9,3)	8,1 (7,8–8,4)^d^
		Ajustada^c^	8,7 (8,4–9,0)	9,8 (9,5–10,2)	9,3 (9,0–9,6)	8,9 (8,6–9,2)	8,1 (7,8–8,4)^d^
	Gordura saturada (% energia total)	Bruta	9,7 (9,6–9,8)	10,4 (10,1–10,6)	10,5 (10,3–10,6)	10,8 (10,6–11,0)	11,8 (11,7–12,0)^d^
		Ajustada^c^	9,8 (9,7–10,0)	10,3 (10,1–10,5)	10,4 (10,3–10,6)	10,8 (10,6–11,0)	11,8 (11,7–12,0)^d^
	Sódio (mg/1000 kcal)	Bruta	1.435 (1.419–1.451)	1.374 (1.358–1.390)	1.409 (1.395–1.422)	1.441 (1.428–1.454)	1.526 (1.508–1.544)^d^
		Ajustada^c^	1.429 (1.414–1.444)	1.377 (1.362–1.393)	1.410 (1.396–1.423)	1.442 (1.429–1.455)	1.526 (1.508–1.545)^d^

a Inclui o consumo de carne bovina *in natura* (carnes e vísceras frescas), processada e alimentos ultraprocessados à base de carne bovina.

b Os percentuais de contribuição da carne bovina para a ingestão total de energia em cada quinto são: Q = 0,0% (0%–0%); Q2 = 3,7% (0,04%–5,7%); Q3 = 7,7% (5,7%–9,8%); Q4 = 12,7% (9,8%–16,0%); Q5 = 23,6% (16,0%–73,4%).

c Médias ajustadas para as diferenças entre os quintos em relação à distribuição das variáveis sexo, idade, escolaridade, renda, região e área.

d p < 0,05 para tendência linear.

Aumentos na participação de carne bovina levam a aumento linear e significativo das pegadas de carbono e hídrica da dieta, sendo essas, respectivamente, três a quatro vezes superiores entre os 20% da população com maior consumo relativo de carne bovina do que entre os 20% com menor consumo.

Aumentos na participação de carne bovina levam a um aumento substancial no teor da dieta em proteína, ferro, zinco e vitamina B_12_ e à diminuição discreta, ainda que significativa, no teor em açúcar de adição. De outro lado, levam a uma redução substancial no teor da dieta em fibra e ao aumento também substancial no teor de gordura saturada e sódio.

A Tabela 4 apresenta estimativas da prevalência de ingestão inadequada de nutrientes na população brasileira e avalia a associação dessa prevalência com a participação de carne bovina na dieta (quintos). A prevalência é muito alta (cerca de 90%) para a insuficiência de fibra e para o excesso de sódio, alta (55,6%) para a ingestão excessiva de gordura saturada e moderada (em torno de 30%) para o excesso de açúcar de adição e para a insuficiência de ferro, zinco e vitamina B_12_. A prevalência da ingestão insuficiente de proteína é baixa (4,2%). Aumentos na participação da carne bovina na dieta levam a reduções substanciais na prevalência da ingestão insuficiente de proteína, ferro, zinco e vitamina B_12_ e a aumentos também substanciais na prevalência da ingestão insuficiente de fibra ou excessiva de gordura saturada e sódio. Não foi observado uma associação estatisticamente significativa entre consumo relativo de carne bovina e a prevalência da ingestão excessiva de açúcar de adição.

**Tabela 4:** Prevalência (%) e razão de *odds* bruta e ajustada (com intervalos de confiança de 95%) para a inadequação na ingestão de nutrientes por quintos da contribuição da carne bovina para a ingestão total de energia. População brasileira com dez ou mais anos de idade, 2008–2009, (n = 32.853).

Nutrientes	Prevalência/Razão de odds (RO)	Quintos da contribuição percentual da carne bovina^a^,^b^					
		Q1	Q2	Q3	Q4	Q5	Total
Proteína	Prevalência	9,1	7,0	2,8	1,4	0,4	4,2
	RO bruta	1 (ref.)	0,75 (0,60–0,95)	0,29 (0,22–0,38)	0,14 (0,10–0,19)	0,04 (0,02–0,07)^d^	
	RO ajustada^c^	1 (ref.)	0,77 (0,60–0,97)	0,30 (0,23–0,38)	0,14 (0,10–0,20)	0,04 (0,02–0,07)^d^	
Fibras	Prevalência	77,1	84,2	84,2	86,8	90,7	84,3
	RO bruta	1 (ref.)	1,58 (1,33–1,88)	1,58 (1,35–1,85)	1,94 (1,65–2,29)	2,89 (2,42–3,46)^d^	
	RO ajustada^c^	1 (ref.)	1,43 (1,20–1,70)	1,45 (1,23–1,71)	1,76 (1,49–2,07)	2,65 (2,20–3,18)^d^	
Ferro	Prevalência	39,8	20,7	23,8	20,7	18,3	25,4
	RO bruta	1 (ref.)	0,39 (0,34–0,45)	0,47 (0,42–0,54)	0,40 (0,35–0,46)	0,34 (0,30–0,39)^d^	
	RO ajustada^c^	1 (ref.)	0,37 (0,32–0,43)	0,45 (0,39–0,52)	0,39 (0,33–0,45)	0,33 (0,29–0,39)^d^	
Zinco	Prevalência	68,1	36,6	30,9	22,5	14,4	35,6
	RO bruta	1 (ref.)	0,27 (0,24–0,31)	0,21 (0,18–0,24)	0,14 (0,12–0,16)	0,08 (0,07–0,09)^d^	
	RO ajustada^c^	1 (ref.)	0,28 (0,24–0,32)	0,21 (0,18–0,24)	0,13 (0,12–0,15)	0,07 (0,06–0,09)^d^	
Vitamina B^12^	Prevalência	58,2	31,8	22,0	14,2	9,4	29
	RO bruta	1 (ref.)	0,33 (0,29–0,38)	0,20 (0,18–0,23)	0,12 (0,10–0,14)	0,07 (0,06–0,09)^d^	
	RO ajustada^c^	1 (ref.)	0,34 (0,30–0,39)	0,20 (0,17–0,23)	0,12 (0,10–0,13)	0,08 (0,06–0,09)^d^	
Açúcar de adição	Prevalência	33,8	43,5	40,6	38,7	34,5	37,9
	RO bruta	1 (ref.)	1,50 (1,32–1,72)	1,34 (1,17–1,52)	1,24 (1,08–1,41)	1,03 (0,89–1,19)	
	RO ajustada^c^	1 (ref.)	1,32 (1,14–1,52)	1,21 (1,05–1,39)	1,10 (0,96–1,26)	0,92 (0,79–1,07)	
Gordura saturada	Prevalência	43,0	48,7	52,8	58,6	76,0	55,6
	RO bruta	1 (ref.)	1,26 (1,10–1,43)	1,48 (1,31–1,68)	1,88 (1,65–2,14)	4,21 (3,63–4,88)^d^	
	RO ajustada^c^	1 (ref.)	1,16 (1,02–1,33)	1,41 (1,24–1,60)	1,83 (1,60–2,08)	4,38 (3,75–5,11)^d^	
Sódio	Prevalência	90,8	88,5	90,6	93,4	96,6	91,8
	RO bruta	1 (ref.)	0,78 (0,64–0,95)	0,99 (0,81–1,20)	1,44 (1,14–1,83)	2,91 (2,26–3,74)^d^	
	RO ajustada^c^	1 (ref.)	0,79 (0,64–0,96)	0,99 (0,81–1,20)	1,43 (1,13–1,82)	2,91 (2,26–3,74)^d^	

a Inclui consumo de carne bovina *in natura* (carnes e vísceras frescas), processada e alimentos ultraprocessados à base de carne bovina.

b Os percentuais de contribuição da carne bovina para a energia total de energia em cada quinto são: Q = 0,0% (0%–0%); Q2 = 3,7% (0,04%–5,7%); Q3 = 7,7% (5,7%–9,8%); Q4 = 12,7% (9,8%–16,0%); Q5 = 23,6% (16,0%–73,4%).

c Razão de *odds* ajustadas para diferenças entre os quintos com relação à distribuição das variáveis sexo, idade, escolaridade, renda, região e área.

d p < 0,05 para tendência linear.

## DISCUSSÃO

Com base no consumo alimentar de uma amostra representativa da população brasileira com dez anos ou mais de idade (n = 32.853)^14^, o presente estudo estimou que a participação de carne bovina na dieta brasileira corresponde a 9,4% da ingestão total de energia. Esta participação é maior entre homens, aumenta com o nível de renda e com a escolaridade e é superior entre os moradores da região Centro-Oeste e de áreas urbanas. As pegadas de carbono e hídrica da dieta aumentam linearmente com quintos da participação da carne bovina na dieta, sendo três a quatro vezes maiores entre os 20% com maior consumo do que entre os 20% com menor consumo. O mesmo aumento no consumo de carne bovina produz resultados mistos na qualidade nutricional da dieta. De um lado, aumenta o seu teor em proteína, ferro, zinco e vitamina B_12_, reduzindo substancialmente a prevalência da insuficiente ingestão desses nutrientes – que é baixa no caso da proteína e moderada no caso dos micronutrientes. De outro lado, reduz o teor da dieta em fibra e aumenta o seu teor em gordura saturada e sódio, aumentando a alta ou muito alta prevalência de ingestão inadequada desses três nutrientes.

Não encontramos na literatura estudos que tenham contrastado a relação que o consumo de carne bovina mantém com as pegadas ambientais da dieta e com sua qualidade nutricional. Há, entretanto, vários estudos que descrevem a associação da pegada de carbono de alimentos ou de padrões alimentares com sua qualidade nutricional, evidenciando em geral um padrão heterogêneo de associação. Uma revisão sistemática desses estudos, em sua maioria realizados em países de alta renda, indica que alimentos e padrões alimentares com baixa pegada de carbono promovem a qualidade nutricional da dieta conforme se associam com menor teor de gordura saturada, porém atuam de modo contrário quando reduzem o teor de micronutrientes como ferro, zinco e vitamina B_12_^9^. Tais resultados são consistentes com os dados do nosso estudo.

Mesmo com tantas variáveis, é possível encontrar dietas que coadunam adequação nutricional e mitigação de impactos ambientais. A dieta mediterrânea e a nova dieta nórdica – ambas com predominância de vegetais e baixo consumo de carnes – são exemplos de padrões alimentares saudáveis que contribuem com a mitigação de impactos ambientais^21^. A adoção dos padrões alimentares recomendados pelo guia alimentar da Holanda oferece as possibilidades de reduzir todas as causas de mortalidade, enquanto reduz moderadamente os impactos climáticos^22^. Estudo da dieta média brasileira (POF 2008/09) ajustada às recomendações do Guia Alimentar para a População Brasileira^14^ mostrou a possibilidade de redução da pegada de carbono em 14,8% e da pegada hídrica em 18,0%^23^.

Medidas de restrição do consumo de carne bovina devem ser feitas cautelosamente, uma vez que esse alimento é fonte de micronutrientes essenciais. Nosso estudo encontrou tendência de aumento no consumo inadequado de ferro, zinco e vitamina B_12_ quanto menor o consumo de carne bovina. Na Holanda, a substituição de 100% de carnes e laticínios por alimentos vegetais na dieta de crianças resultou em diminuição de 5% a 13% na ingestão de cálcio, zinco e vitamina B_1_ e de 49% na ingestão de vitamina B_12_, além de aumentar a ingestão de ferro com menor biodisponibilidade^24^.

A relação entre o consumo de carne bovina (e de outros alimentos de origem animal) e o teor de micronutrientes na dieta serve de um alerta para que a qualidade nutricional da alimentação seja sempre levada em conta na caracterização das dietas ambientalmente sustentáveis. Para que as recomendações de redução do consumo de carne bovina tenham consistência com as metas de mitigação dos impactos climáticos, os desafios nutricionais não podem ser negligenciados.

A intensidade do impacto ambiental dos alimentos é um entre vários critérios a serem usados nas escolhas alimentares. A qualidade nutricional dos alimentos que compõem a fração vegetal das dietas é importante, pois nem todos os alimentos de origem vegetal têm efeitos cardiovasculares benéficos ou, ainda, alguns deles podem ser prejudiciais à saúde, como é o caso dos alimentos ultraprocessados de origem vegetal^25^. Isso significa que tais alimentos devem ser evitados independentemente de ter potencial de impacto ambiental alto ou baixo.

A redução do consumo de carne bovina pela população brasileira é uma estratégia-chave na mitigação dos impactos climáticos no país, mas sua efetividade tem limites. Ainda que a participação da carne bovina na dieta dos brasileiros diminua, o Brasil é o principal fornecedor de carne bovina no mercado internacional. A sustentabilidade ambiental do sistema alimentar não pode depender simplesmente de mudanças na demanda interna pela carne bovina; eventuais resultados ambientais positivos alcançados pelo consumo interno menor poderiam ser anulados pelo aumento no volume de exportação de carne bovina ou, ainda, por interrupções no abate^26–27^. As transformações na produção pecuária que resultem em sistemas de produção de impactos ambientais baixos são, portanto, urgentes.

Os pontos fortes desse estudo incluem o uso de amostra representativa da população brasileira, a mensuração do consumo alimentar por meio do registro de todos os alimentos consumidos pelas pessoas em dois períodos de 24 horas e a avaliação do impacto do consumo de carne bovina tanto sobre o meio ambiente quanto sobre a qualidade nutricional da dieta, possibilitando avaliações das dietas em situações reais de consumo nos diferentes estratos populacionais. Este estudo focalizou a carne bovina, um alimento relevante na cultura culinária nacional, cujo setor produtivo é responsável por cerca de 25% das emissões de gases do efeito estufa contabilizadas no inventário nacional^28^. A avaliação abrangente da qualidade nutricional das dietas paralelamente às medidas de consumo inadequado de nutrientes críticos e outros que devem ser limitados foi uma inovação da pesquisa.

Uma das principais limitações desse estudo diz respeito ao uso de coeficientes de pegadas de carbono e hídrica calculados nem sempre com base nas condições de produção observadas no Brasil. Outra limitação, comum aos inquéritos nutricionais, é a imprecisão na quantificação do consumo de alimentos e de nutrientes decorrente do autorrelato do consumo alimentar e do uso de tabelas de composição nutricional de alimentos que nem sempre traduzem a composição dos alimentos ingeridos pelos participantes. As pegadas ambientais dos produtos ultraprocessados à base de carne bovina foram consideradas para o produto como um todo, constituído de outros ingredientes de origem vegetal e aditivos, e não apenas para a parcela de carne bovina. Finalmente, a não inclusão de menores de dez anos na amostra estudada impossibilita a generalização de resultados para essa faixa etária.

Outra limitação deste estudo é basear-se em um inquérito de consumo alimentar realizado há mais de dez anos. Entretanto, comparando-se o consumo estimado pela POF realizada em 2008/09^14^ com o estimado pela POF mais recente de 2017/18^29^, observam-se mudanças de muito pequena magnitude, inclusive quanto ao consumo de carne bovina: 63 g/pessoa/dia e 60 g/pessoa/dia, respectivamente.

Este trabalho procurou apontar algumas influências nutricionais e ambientais do consumo de carne bovina nas dietas brasileiras. Os quadros recentes de redução do consumo de carne bovina, insegurança alimentar e fome resultantes do empobrecimento da população brasileira – concomitantes aos recordes de produção de alimentos e de desmatamento –, só confirmam a insustentabilidade do sistema alimentar no Brasil. Reiteramos que um sistema alimentar sustentável pressupõe a segurança alimentar da população e deve ser avaliado nas várias dimensões do desenvolvimento sustentável: social, cultural, ambiental, ecológica, territorial, econômica e política^30^.

## CONCLUSÃO

A redução no consumo de carne bovina no Brasil tenderia a diminuir as pegadas de carbono e hídrica da dieta e o risco de doenças crônicas relacionadas à alimentação, mas, para não aumentar o risco de deficiências nutricionais, a redução teria que ser acompanhada do aumento na ingestão de outros alimentos fontes de proteína, ferro, zinco e vitamina B_12_.
